# A New Classification of the Severity of Psoriasis: What’s Moderate Psoriasis?

**DOI:** 10.3390/life11070627

**Published:** 2021-06-29

**Authors:** Laura Salgado-Boquete, José Manuel Carrascosa, Mar Llamas-Velasco, Ricardo Ruiz-Villaverde, Pablo de la Cueva, Isabel Belinchón

**Affiliations:** 1 Dermatology Department, Complejo Hospitalario Universitario de Pontevedra, 36003 Pontevedra, Spain; Laura.salgado.boquete@sergas.es; 2 Dermatology Department, Hospital Universitari Germans Trias I Pujol, Universitat Autònoma de Barcelona, IGTP, 08916 Badalona, Spain; 3 Dermatology Department, Hospital Universitario de La Princesa, 28006 Madrid, Spain; Mariadelmar.llamas@salud.madrid.com; 4 Dermatology Department, Hospital Universitario San Cecilio, Instituto Biosanitario de Granada, Ibs, 18016 Granada, Spain; Ricardo.ruiz.villaverde.sspa@juntadeandalucia.es; 5 Dermatology Department, Hospital Universitario Infanta Leonor, 28031 Madrid, Spain; pcueva@salud.madrid.org; 6 Dermatology Department, Hospital General Universitario de Alicante-ISABIAL-UMH, 03010 Alicante, Spain; belinchon_isa@gva.es

**Keywords:** psoriasis, severity, classification, moderate, discrimination, cut-offs

## Abstract

The purpose of this study is to propose a ranking system for the severity of psoriasis. The consensus method of selecting the indices to include and the classification of real patient profiles by an expert panel to create a gold standard of severity were used. The performance of potential cut-offs was evaluated to create a ranking algorithm. The combined use of PASI, BSA, and sPGA may allow the classification of the severity of psoriatic patients. The final algorithm identifies severe patients in a single step (2 out 3 are met: PASI ≥ 11 or BSA ≥ 10 or sPGA ≥ 3), while two steps are required for mild ((2 out 3 are met: PASI ≤ 3 or BSA ≤ 5 or sPGA ≤ 2) and DLQI < 5) and moderate forms (the patient does not meet 2 out 3 (PASI ≥ 11 or BSA ≥ 10 or sPGA ≥ 3) but has a DLQI ≥ 5. A ranking algorithm is presented, consisting of different measures of disease which classifies psoriatic patients into three categories: mild, moderate, and severe.

## 1. Introduction

Psoriasis is a chronic, multisystem inflammatory disease, which primarily affects the skin, with a prevalence of around 2–5% [1]. Its aetiology is a complex interplay between genetics, environment, skin barrier disruption, and immune dysfunction [2,3]. As a systemic process, psoriasis is associated with a high degree of comorbidity [4], while anti-psoriasis medications can be associated with significant undesirable effects. Disease severity can be instrumental in guiding treatment decisions and defining eligibility criteria for clinical studies. In clinical practice, psoriasis severity assessment usually combines objective and subjective parameters, including the extent of skin involvement, location of lesions, thickness, symptoms, and the disease’s impact on patients’ lives [5,6].

Psoriasis severity assessment has been typically based on a dichotomous classification comprising mild and moderate-severe forms. The difference between both forms is based on various clinical scales, among which the “rule of 10” is one of the most accepted, probably because of its simplicity and ease of use [7]. According to this rule, patients with Psoriasis Area Severity Index (PASI), body surface area (BSA), and Dermatology Life Quality Index (DLQI) values below 10 would be considered to display mild psoriasis, while those with values above 10 in any of these scales would be deemed to exhibit moderate to severe psoriasis. This classification appears problematic because it relies on subjective scales designed to avoid under-treatment in low PASI cases, while involving specific areas like the palmoplantar or facial, thereby generating a severe disease burden. On the other hand, patients with moderate-severe psoriasis will be targeted for systemic biological treatment [8,9].

Although this dichotomous classification of psoriasis severity has been well established, proposals for a further classification of moderate forms have emerged in recent years, in an attempt to better differentiate this category from both severe and mild psoriasis forms. To this end, the 2011 guidelines of the American Academy of Dermatology (AAD) defined three disease categories, which are primarily based on BSA [10], considering moderate psoriasis patients as having a BSA between 5% and 10%, defining a BSA below 5% as mild psoriasis, and referring to a BSA of 10% or more as severe psoriasis.

In 2017, Llamas-Velasco et al. published a proposal for defining moderate psoriasis [11] based on the opinion of six expert dermatologists from the psoriasis group of the Spanish Academy of Dermatology and Venereology (AEDV). The categories of mild, moderate, and severe were defined according to PASI and DLQI. These three disease categories were defined as follows: (a) mild psoriasis if PASI < 7 and DLQI < 5; (b) moderate psoriasis if PASI < 7 and DLQI ≥ 5; (c) severe psoriasis if PASI > 15 with any DLQI value. Therefore, this latter classification’s main limitation is that it is solely based on an expert consensus. Consequently, this classification should not be considered a definition but rather a proposal that could serve as a starting point for further development using an appropriate methodology.

In 2018, Knuckles and collaborators attempted to better define the moderate psoriasis concept, as based on surveys focused to North American dermatologists [12]. Although the majority of dermatologists stated that they employed BSA to assess psoriasis severity, the high variability observed demonstrated the lack of consensus concerning the definition of moderate psoriasis. Therefore, there is currently no agreement on the concept of moderate psoriasis, despite several other proposals published in the scientific literature [13].

This work primarily sought to propose a classification system for psoriasis severity and to evaluate its performance in classifying psoriatic patients into three categories: mild, moderate, and severe.

## 2. Methods

A two-phase study was developed. First, a Delphi survey was conducted, which primarily aimed at selecting the indices to be included in the classification system. In a second step, a specific methodology was applied to generate a ranking system based on patient profiles.

### 2.1. Expert Panels

The experts were selected from dermatologists of the AEDV psoriasis group with sufficient representation from large and small centres, geographic areas, and both genders. The steering group was composed of six dermatologists and two methodologists with experience in outcome development. The steering group was instrumental in designing the surveys, providing the profiles, and classifying the patients into these profiles. In addition, the full AEDV psoriasis group (*n* = 73) was invited to participate in the Delphi rounds.

### 2.2. Selection of Indices to Be Included in the Proposal

The Delphi survey was designed to gather the opinions of dermatologists with expertise in psoriasis concerning the indices that should be included in the severity classification. The degree of agreement was obtained through 5-level Likert responses, ranging from ‘1 = No agreement’ to ‘5 = Full agreement’ in anonymous rounds. In the first round, items voted in favour (4 or 5) by more than 80% of the dermatologists were retained, while those agreed upon by 20% or fewer were deleted. Items with intermediate scores (>20% and <80%) were subjected to a second round. After the last round, all those items attaining over 80% were accepted.

### 2.3. Patient Profiles and Gold Standard

Patient profiles (346 cases from six reference hospitals) were generated by compiling retrospective real data in an anonymized way. Each patient profile was descripted by its scores on the chosen indices. These profiles were then classified based on consensus among the steering expert panel (gold-standard) into the three severity levels, mild, moderate, and severe. The pre-planned number of patients for classification and assessment by ROC curves was 300, given the expected percentages of the different psoriasis severity categories (assuming 70% and 30% for mild and moderate-severe forms and AUC = 0.70).

### 2.4. Statistical Analysis, Definitions, and Diagnostic Performance

Once the patients were classified into one of the three severity categories, two subgroups were created, comprising mild versus (vs.) moderate forms, and moderate vs. severe forms. In each of these two subgroups, an array of analyses was performed.

Comparison between severity subgroups. The subgroups were described in terms of the chosen indices using descriptive statistics and compared using parametric (Student’s t) or non-parametric (Mann–Whitney U) tests, depending on the respective distributions.

Contribution of the indices to the classification. A bivariate logistic regression analysis was carried out to assess the association strength of each of the indices selected by the Delphi study with the classification variable of subgroups (mild vs. moderate and moderate vs. severe).

Cut-off points and performance. As possible cut-off points, statistical parameters were calculated for each index, both in the mild and severe forms. For the mild forms, the 90th and 95th percentiles were considered, while for the severe forms, the 5th and 10th percentiles were chosen. In order to enable the classification of patients, the cut-off points for some scores had to be integers, and as a result, some cut-offs were rounded-up. The correct classification percentage according to the gold standard based on the 90th percentile of each index was then calculated.

The number of indices that could not exceed these cut-offs so as to be classified as mild or severe psoriasis was then investigated, generating potential operational definitions [13,14]. The performance of each definition was then evaluated using ROC curves with the expert’s classification as gold standard. Sensitivity, specificity, and area under the curve (AUC) were then calculated. The best definition according to these parameters was finally selected.

All analyses were performed using Stata 12 statistical software (Stata Corporation, College Station, TX, USA).

## 3. Results

### 3.1. Selection of Indices to Be Included in the Definition: Delphi Study

The Delphi study was answered by 73 dermatologists (89% of the AEDV psoriasis group). The indices with an agreement over 80% were PASI, BSA, and DLQI. The indices with an intermediate agreement (20–80%) were included in the second round, as follows: static Physician Global Assessment (sPGA), Scalp Physician Global Assessment (ScPGA), static Physician Global Assessment of Genitalia (sPGA-G), palmoplantar psoriasis Physician Global Assessment (ppPGA), fingernail Physician Global Assessment (fPGA), and itching visual analogue scale (VAS). Of the 73 dermatologists who participated in the first round, 62 responded to the second (response rate 84.9%). In the second round, sPGA and itching VAS had agreements over 80% and were thus added to PASI, BSA, and DLQI for the next phase.

### 3.2. Patient Profiles

A sample of 349 patients was obtained. Significant variability was observed in the classification of these patients by the dermatologists, with individual results from 29.5% to 51.3% for mild psoriasis, 29.2% to 44% for moderate, and 13.2% to 41.3% for severe. The classification was established by evaluator agreement ≥80%. As there were six expert dermatologists, the 80% agreement was set on a concordant assessment among five of them. Consequently, 116 patients (45.7%) were classified as mild, 70 (32.1%) as moderate, and 44 (20.2%) as severe. The total number of patients classified was 254 (72.8% of the sample). This consensus classification was used as the gold standard to evaluate the potential definitions’ performances. The differences across indices between mild versus moderate and moderate versus severe forms were all statistically significant.

### 3.3. Strength of Association

Bivariate logistic regression analysis showed that all indices were associated with both classification groups. Increases in PASI, BSA, DLQI, sPGA and itching VAS scores increased the probability of being classified as having moderate and severe psoriasis vs. mild and moderate. This was true for all indices except for PASI in the discrimination between moderate and severe forms. Similarly, the DLQI value to differentiate between mild and moderate patients showed an important association (OR = 1.8), whereas this association was weaker in regards to the moderate-severe differentiation (OR = 1.1).

### 3.4. Cut-Off Points

Table 1 displays the summary statistics that were tested as cut-offs. The classification percentage of each severity category of the gold standard variable was calculated according to the 90th percentile of each index (Table 2). The percentage of correct classification was good (>75%) for all indices except for DLQI and pruritus VAS, which classified correctly only 26% and 11% of the severe forms, respectively. This was also obvious by looking at the distribution of the summary statistics with wide overlap between the mild and severe forms for these two indices. Therefore, subsequent steps included only the physician-derived indices (PASI, BSA and sPGA), treating patient-derived indices as complementary information. Given that the itching VAS classified correctly only very few patients and that a pruritus measure is already included in the DLQI, the itching VAS was omitted from subsequent working definitions.

### 3.5. Models and Performance: ROC Curves

**Figure 1:**
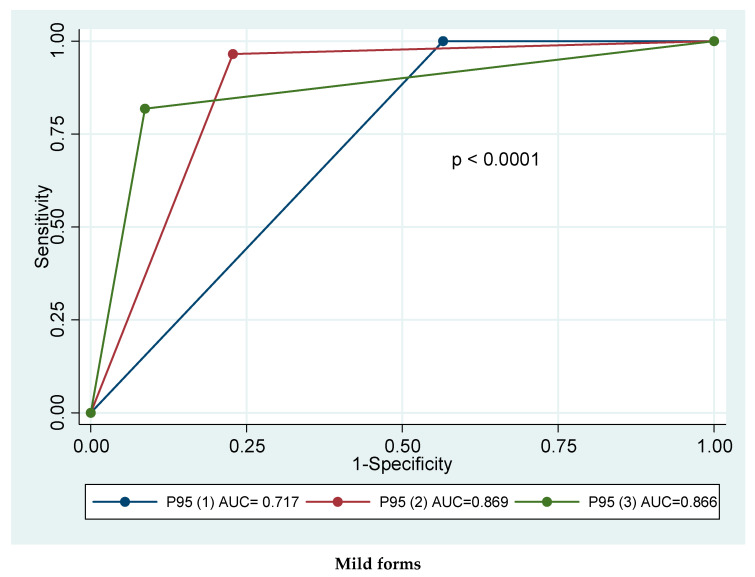

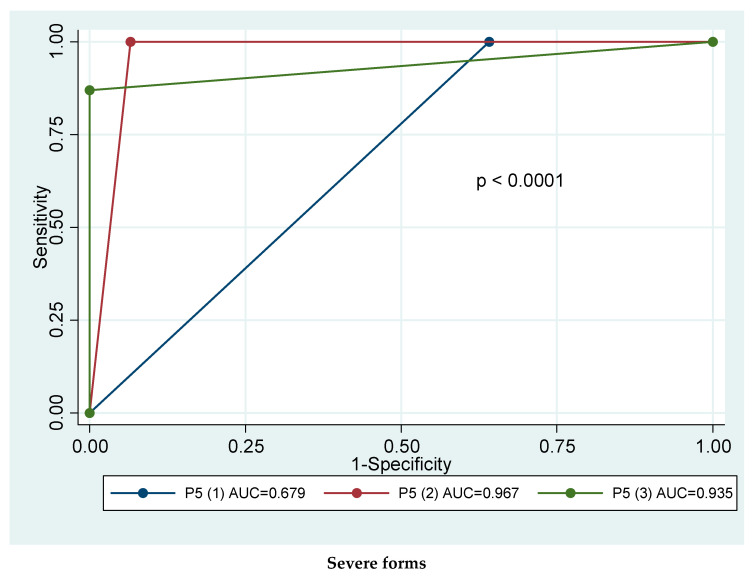
ROC curves for candidate definitions.

The models to be tested were created by variations of the three indices (PASI, BSA, and sPGA) in the number of statistical parameters met by each patient. For mild forms, values of each index lower than or equal to the 95th percentile of this category were used as cut-offs, thus generating three proposals. For severe forms, values higher than or equal to the 5th percentile in the three indices were used, which again generated three proposals. Table 3 shows the performance of each definition, depending on the number of criteria met. The best proposal for mild psoriasis met at least two criteria, corresponding to the 95th percentile of the three physician-derived indices (sensitivity 96%; specificity 77%; AUC 0.869). Comparison of the ROC curves of these approaches revealed statistically significant differences (*p* < 0.0001). For severe psoriasis, the best possibility corresponded to the one that met at least two criteria (S = 100%; E = 93%; AUC = 0.967). The difference between curves was statistically significant (*p* < 0.0001) (Figure 1).

Therefore, while using only physician-derived variables, the best approach for mild psoriasis would be to score below the 95th percentile on at least two of the three indices considered, i.e., two out of PASI ≤ 3, BSA ≤ 5, or sPGA ≤ 2.

Likewise, the best proposal for severe psoriasis would be the one that attained at least two indices over the 5th percentile, i.e., two out of PASI ≥ 11, BSA ≥ 10, or sPGA ≥ 3.

**Figure 2:**
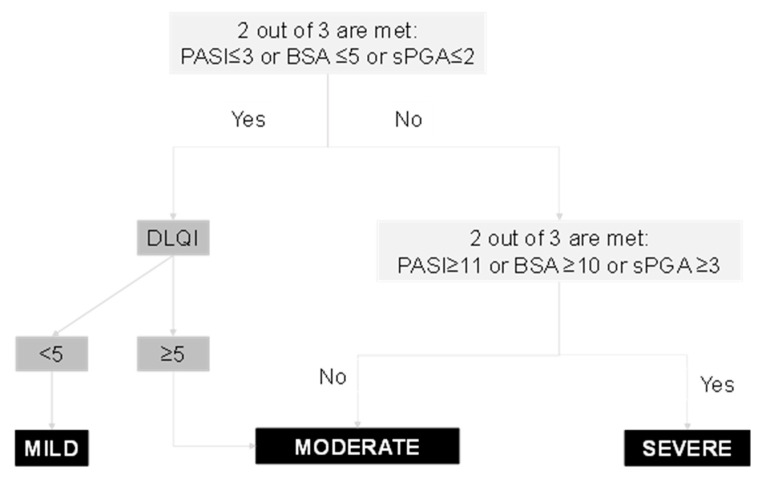
Proposal of a psoriasis classification severity algorithm.

Given that the DLQI was initially selected as an index to be included in the severity definition, we have proposed an algorithm to include patient-derived information into the ranking system. In the DLQI case, a value equal to or less than P90 (4) correctly classified 92.2% of the mild forms, while the higher values (between 5 and 20) classified 74% of the moderate forms (Table 2). These results enabled us to apply cut-off point of = 5 for the DLQI in order to correctly classify patients with mild psoriasis forms as the first step of the proposed algorithm (Figure 2).

## 4. Discussion

This study aimed to establish a ranking system of psoriasis severity into three categories consisting of mild, moderate, and severe. We obtained a ranking proposal with face and content validity supported by real patients’ data. The system classifies patients according to three objective measures including PASI, BSA, and sPGA. The final product is an algorithm that enables severe patient classification in a single step, with two steps required for mild and moderate forms. Psoriasis patients are considered severe if they present at least two of the following: PASI ≥ 11, BSA ≥ 10, and sPGA ≥ 3. In contrast, if a patient displays at least two of the following: PASI ≤ 3, BSA ≤ 5, and sPGA ≤ 2, then a second step must be applied based on DLQI score. If DLQI is <5, the patient is classified as mild and if it is ≥5, the patient is classified as moderate.

A severity classification of psoriasis patients is primarily needed to guide healthcare decisions and research [2,5,15]. Psoriasis disease displays essential heterogeneity in its clinical expression and treatment response, with disease duration, areas of involvement, as well as percentage of body surface and other factors being contributors. Besides, severity and impact may fluctuate throughout a life span, and an individual patient may thus be classified differently at various points of the disease course. Targeted therapies and therapeutic goals in psoriasis warrant clear indications, criteria, and definitions as well [16,17,18].

The terms ‘mild’, ‘moderate’, and ‘severe’ psoriasis are widely used, despite the absence of agreed upon criteria for defining them [5]. Various severity classification systems already exist, whereas none have reached a wide consensus, nor do any allow for a clear separation between moderate and severe forms, although such a difference may be of great relevance when choosing therapeutic options [18,19]. To date, the methodology employed to establish psoriasis severity classifications is solely based on consensus methods [6,18,20].

This severity ranking obtained in our case is very simple. Moreover, the system contains both measures that are widely used by dermatologists (PASI, BSA, and sPGA), as well as those taking the patient’s perspective (DLQI) in account, with all of them being widely accepted. The three physician-derived indices may contain overlapping information and, therefore, only two of the three indices are required for the classification. This would allow for its application in settings where only BSA or PASI are employed, as long as a Physician Global Assessment is added to them. The inclusion of a quality-of-life measure is paramount. Indeed, severity is inextricably linked to impact on quality of life [5,21,22]. Therefore, the combination of both perspectives into a severity ranking system renders the proposal more complete and closer, as well, to the clinical reality of psoriatic patients.

A strength of our study is its use of a mixed methodology, with consensus techniques applied to define the indices to be included, in addition to patient profiling and robust statistical methods, with the aim of analysing the performance of different combinations against a gold standard. This mixed methodology was based on more than expert opinions, unlike as usually done in the past [11,12,19,23], thus avoiding the potential bias of consensus methods. Moreover, this mixed procedure has already been used to define other psoriasis constructs, such as ‘minimal disease activity’ [13,24]. The Delphi survey results confirmed the relevance of five indices (PASI, BSA, sPGA, Itching VAS, and DLQI). Interestingly, the steering group preferred indices than rather domains, as is usually the case in these studies [14,24,25]. Indeed, this was a way of speeding up the process and was based on previous experiences of the AEDV psoriasis group [24]. However, the results do not differ much from other initiatives where patients were included to reach a definition of disease activity [14,24]. The proposals then underwent then a second validation phase against a gold standard. The absence of a good gold standard, as pointed out by others [23], renders the validation objective more difficult, and we cannot rule out a certain circularity in our classification variable. It was notable that the variability among experts in classifying the patients actually reflected the arbitrariness in the existing definitions. This underscores the need to establish a clear classification with measurable criteria in order to avoid every dermatologist having a different definition in mind. Our methods went further by testing cut-offs based on summary statistics rather than arbitrary cut-offs. By doing so, the itching scale was found not to discriminate severe psoriasis, and it was therefore removed from the definition. Concerning DLQI, discrimination of severe patients was similarly low (26%), yet it was maintained owing to the disease’s impact’s relevance, especially its ability to distinguish between mild and moderate forms, and also because it includes an itching measure, which was found to be important to start with.

As expected, a definition of ‘moderate’ psoriasis cannot be achieved in a single step, but only after ruling out mild and severe psoriasis forms. This is probably the reason why previous experiences and treatment guidelines have avoided mentioning ‘moderate’ psoriasis and have, instead, provided definitions that merged ‘moderate’ and ‘severe’. Without clearly delimiting the borders of mild and severe, as we did with the percentile divisions, we could not have considered the middle areas. The target question is how important it is to reach a definition of moderate psoriasis. Some groups advocate the need of an intermediate treatment level located in between what is recommended for mild and severe psoriasis, possibly reflecting that severity seems to be driven by indications [5,19].

This study has its limitations. Firstly, the proposed system does not include the relevance of special locations. This information could only be indirectly captured through the DLQI score; as a result, we could only classify these patients into either mild or moderate forms.

In conclusion, a classification algorithm for psoriasis has been presented, consisting of measures of disease burden, both from the dermatologists’ and patients’ perspectives, which enables us to classify patients into three categories: mild, moderate, and severe. While this clear algorithm is based on mixed methodology, it still needs to be validated in cohort studies.

The increased understanding of psoriasis as a systemic disease makes the study of subpopulations particularly relevant, not only for therapeutic purposes, but also for the management of the disease and associated comorbidity. Differentiation between moderate and severe disease can be useful to select more homogeneous patient populations for clinical and research purposes. In this sense, the use of the proposed classification in future studies would allow the analysis of whether there are differences in treatments efficacy or the incidence or the impact of comorbidities in different subgroups of the disease severity spectrum.

## Figures and Tables

**Table 1:** Potential cut-off points of the chosen indices to include in the definition of mild and severe psoriasis.

	Index	P_5_	P_10_	P_90_	P_95_	Min	Max
**Mild Psoriasis**	**PASI**	0.3	0.6	3.5	3.8	0	5.6
	**BSA**	0.2	0.4	4	5	0	7
	**sPGA**	1	1	2	2	0	3
	**DLQI**	0	0	4	5	0	17
	**Itching VAS**	0	0	5	7	0	8
**Severe Psoriasis**	**PASI**	11	12	31.2	35	10	44
	**BSA**	10	11	63.5	66	8	90
	**sPGA**	3	3	4	4	2	4
	**DLQI**	1	4	25	25	1	29
	**Itching VAS**	3	4	10	10	0	10

Abbrev. min, minimum; max, maximum.

**Table 2:** Percentage of correct classification according to P90.

	Mild (*n* = 116)	Moderate (*n* = 92)	Severe (*n* = 46)
**PASI ≤ 3**	97 (83.6%)	11 (11.9%)	-
**PASI 4–8**	26 (22.4%)	75 (81.5%)	-
**PASI > 8**	-	10 (10.9%)	46 (100%)
**BSA ≤ 4**	106 (91.4%)	18 (19.6%)	
**BSA 5–9**	9 (0.08)	60 (65.2%)	2 (4.3%)
**BSA > 9**		11 (11.9%)	44 (95.6%)
**sPGA ≤ 2**	113 (97.4%)	36 (39.1%)	2 (0.04%)
**sPGA 2–3**	43 (37.1%)	81 (88.0%)	18 (39.1%)
**sPGA > 3**		4 (0.04%)	28 (60.9%)
**DLQI ≤ 4**	107 (92.2%)	16 (17.4%)	5 (10.9%)
**DLQI 5–20**	9 (0.07%)	68 (73.9%)	29 (63.0%)
**DLQI > 20**		8 (8.7%)	12 (26.1%)
**Itching VAS ≤ 5**	107 (92.2%)	51 (0.55%)	13 (0.28)
**Itching VAS 6–9**	9 (0.07)	31 (33.7%)	24 (52.2%)
**Itching VAS > 9**		6 (0.06%)	5 (10.9%)

Abbreviations: PASI, Psoriasis Area Severity Index; BSA, body surface area; DLQI, Dermatology Life Quality Index; sPGA, static Physician Global Assessment; VAS, visual analogue scale.

**Table 3:** Performance of the different definitions.

	No. Criteria Met *	Sensitivity	Specificity	AUC (95% CI)
**Mild Psoriasis**	≥1	100	43.5	0.717 (0.650–0.776)
	≥2	96.5	77.2	0.869 (0.817–0.913)
	3	81.9	91.3	0.866 (0.811–0.909)
**Severe Psoriasis**	≥1	100	35.9	0.679 (0.596–0.758)
	≥2	100	93.5	0.967 (0.927–0.992)
	3	87.0	100	0.935 (0.880–0.970)

* Out of PASI, BSA, and sPGA and based on the P_95_ in the indices for mild forms and P_5_ for severe forms.

## Data Availability

Not available.
